# Essential Oil of *Aristolochia trilobata*: Synthesis, Routes of Exposure, Acute Toxicity, Binary Mixtures and Behavioral Effects on Leaf-Cutting Ants

**DOI:** 10.3390/molecules22030335

**Published:** 2017-02-25

**Authors:** Bruna Maria S. de Oliveira, Carlisson R. Melo, Péricles B. Alves, Abraão A. Santos, Ane Caroline C. Santos, Alisson da S. Santana, Ana Paula A. Araújo, Pedro E. S. Nascimento, Arie F. Blank, Leandro Bacci

**Affiliations:** 1 Departamento de Engenharia Agronômica, Universidade Federal de Sergipe, São Cristóvão 49100-000, Brazil; bruna_barreiros02@hotmail.com (B.M.S.d.O.); carlisson_melo@hotmail.com (C.R.M.); abraaoufs@gmail.com (A.A.S.); aneccelestinos.18@gmail.com (A.C.C.S.); alisson.0910.silva@gmail.com (A.d.S.S.); arie.blank@gmail.com (A.F.B.); 2 Departamento de Química, Universidade Federal de Sergipe, São Cristóvão 49100-000, Brazil; periclesbalves@gmail.com (P.B.A.); pedro_ellison@hotmail.com (P.E.S.N.); 3 Departamento de Ecologia, Universidade Federal de Sergipe, São Cristóvão 491000-000, Brazil; anatermes@gmail.com

**Keywords:** Aristolochiaceae, insecticidal plant, monoterpenes, Formicidae, fumigation, synergism, additive effect, repellency

## Abstract

Plants of the genus *Aristolochia* have been frequently reported as important medicinal plants. Despite their high bioactive potential, to date, there are no reports of their effects on leaf-cutting ants. Therefore, the present study aimed to evaluate the insecticidal activity of the essential oil of *Aristolochia trilobata* and its major components on *Atta sexdens* and *Acromyrmex balzani*, two species of leaf-cutting ants. The bioassays were performed regarding routes of exposure, acute toxicity, binary mixtures of the major components and behavioral effects. Twenty-five components were identified in the essential oil of *A. trilobata* using a gas chromatographic system equipped with a mass spectrometer and a flame ionization detector. The components found in higher proportions were sulcatyl acetate, limonene, *p*-cymene and linalool. The essential oil of *A. trilobata* and its individual major components were efficient against *A. balzani* and *A. sexdens* workers when applied by fumigation. These components showed fast and efficient insecticidal activity on ants. The components acted synergistically and additively on *A. balzani* and *A. sexdens*, respectively, and caused a strong repellency/irritability in the ants. Thus, our results demonstrate the great potential of the essential oil of *A. trilobata* and its major components for the development of new insecticides.

## 1. Introduction

Leaf-cutting ants (Hymenoptera: Formicidae) stand out as one of the most important pests in Neotropical regions because of their high abundance and wide distribution and the enormous economic losses they cause to agriculture and forestry [1]. One of the factors responsible for the population growth of leaf-cutting ants in disturbed areas is the increased density near forest edges of pioneer plants, i.e., plants low in chemical defenses, emphasizing the importance of secondary plant metabolites in regulating herbivore populations. In fact, plant chemical defenses have been suggested as more promising components for the management of leaf-cutting ants than synthetic, organic insecticides [2] because the latter cause diverse negative effects [3,4], increasing the search for environmentally safer and more efficient compounds in recent years. Plant essential oils stand out in this context because they have shown toxicity by different routes of exposure and have caused several behavioral effects on pest insects [5].

The essential oils of plants include several types of chemicals, with most of them consisting of low-molecular-weight mono- and sesquiterpenes. One of the advantages of essential oils is that they consist of complex mixtures of various chemical components, thus decreasing the probability of insect resistance development. Other advantages of using these products include their rapid degradation in the environment, fast effect on the target organism and relatively low toxicity to mammals [6]. Another notable characteristic of essential oils compared with other products, such as conventional insecticides, is the interaction between their components. When synergistic, these interactions may potentiate the biological effects of essential oils, allowing the application of lower doses and, consequently, reducing management costs and environmental risks [7]. These synergistic actions may occur between major essential oil components, thus increasing the oil efficacy [8,9,10].

The family Aristolochiaceae Juss. comprises approximately 600 plant species distributed in subtropical, tropical and temperate regions [11]. These species are frequently reported as important medicinal plants [12]. Extracts from different species of *Aristolochia* have shown bioactivity against insects [12,13]. However, the potential use of the essential oils of plants belonging to the family Aristolochiaceae Juss. for pest control has not been investigated.

The present study evaluated the insecticidal activity of *Aristolochia trilobata* against workers of the leaf-cutting ants *Acromyrmex balzani* and *Atta sexdens* with the following aims: (i) identifying and quantifying the components present in *A. trilobata* essential oil; (ii) studying the route of exposure of these insects to the essential oil and its major components; (iii) evaluating the toxicity of the essential oil and its major components to *Acromyrmex balzani* and *Atta sexdens* workers; (iv) determining the presence of synergistic, additive and/or antagonistic effects after the binary mixing of major essential oil components; and (v) observing the behavioral responses of ant workers exposed to the essential oil and its major components. Based on the results of the present study, the essential oil of *A. trilobata* and its major components—sulcatyl acetate, limonene, *p*-cymene and linalool—showed insecticidal activity against *A. balzani* and *A. sexdens* when applied by fumigation, affecting the survival and behavior of these species.

## 2. Results and Discussion

### 2.1. Essential Oil Characterization

A total of 25 components, representing 98.72% of the total composition of *A. trilobata* essential oil, were identified by a gas chromatographic system equipped with a mass spectrometer and a flame ionization detector (GC/MS/FID), with the majority of the components belonging to the monoterpene family (84.83%) (Table 1).

**Figure 1:**
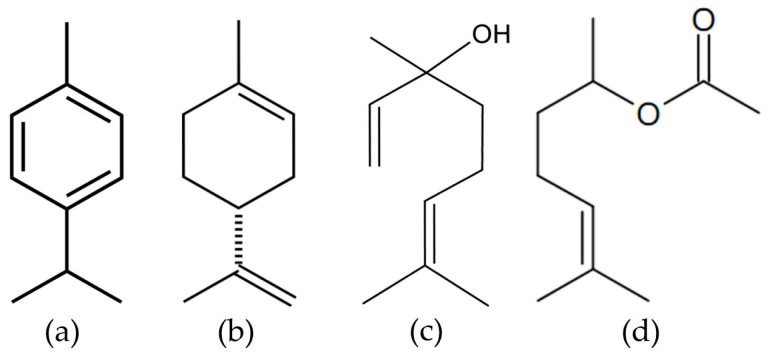
Four major components found in the essential oil of *Aristolochia trilobata*: (**a**) *p*-cymene; (**b**) limonene; (**c**) linalool; and (**d**) sulcatyl acetate.

The most abundant monoterpenes included sulcatyl acetate (25.64%), limonene (24.80%), *p*-cymene (10.41%) and linalool (9.51%) (Figure 1). In all, 13 components were found in concentrations below 1%, and eight components ranged in concentration from 1% to 6% (Table 1). The aristolochic acids, which are commonly found in plants of the genus *Aristolochia* [14], were not detected in the present study. Studies have shown that aristolochic acids have nephrotoxic, carcinogenic and mutagenic effects [15,16,17].

The major components found in the essential oil of *A. trilobata* (sulcatyl acetate, limonene, *p*-cymene and linalool) have all been previously reported [19], although their concentrations were higher in the present study. Such variation is common, being determined by genetic and/or environmental factors [8,20]. Sulcatyl acetate, the major component, has been considered a marker for *A. trilobata* [19]. Components or classes of components can be considered markers for a plant species when there is correlation with a biological effect [21].

### 2.2. Routes of Exposure Bioassays

**Figure 2:**
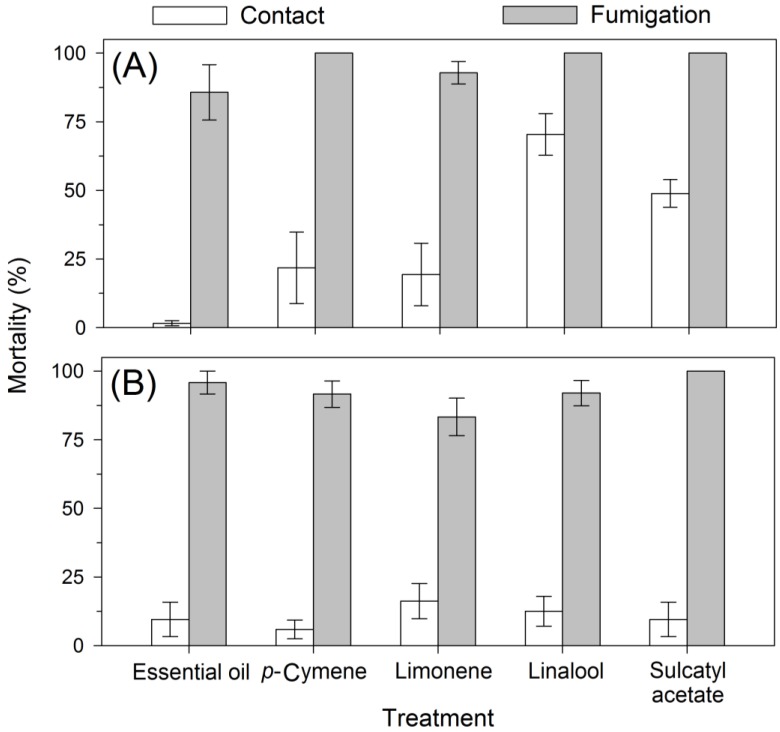
Toxicity of *Aristolochia trilobata* essential oil and its major components to workers of *Acromyrmex balzani* (**A**) and *Atta sexdens* (**B**) 48 h after exposure by topical application (10 μg·mg^−1^) and fumigation (10 μL·L^−1^).

The essential oil of *A. trilobata* and its major components were effective against *A. balzani* and *A. sexdens* workers when the route of exposure was fumigation. These components caused greater than 80% mortality after 48 h of exposure via fumigation. When the components were applied topically, mortality was less than 70% and 20% for *A. balzani* and *A. sexdens*, respectively (Figure 2).

*Pogostemon cablin* essential oil shows contact toxicity for the ant species *Camponotus melanoticus, Camponotus novogranadensis* and *Dorymyrmex thoracicus* [22]; however, contact toxicity was not observed in the present study. Therefore, the absence of toxicity of *A. trilobata* essential oil and its major components to *A. balzani* and *A. sexdens* when topically applied possibly indicates that these components did not penetrate the exoskeleton in lethal amounts when applied by this exposure route. This barrier to penetration may be determined by the chemical composition of the insect cuticle and/or by the physicochemical properties of the components present in the essential oil [8,23].

By contrast, all the components analyzed in the present study showed a strong effect when applied by fumigation. The scarcity of studies on the fumigation toxicity of essentials oils to ants prevents further comparisons. However, the essential oil components clearly have potential as fumigants because they caused mortality above 80% [24].

### 2.3. Acute Fumigation Toxicity

The essential oil of *A. trilobata* showed efficient insecticidal activity against both *A. balzani* and *A. sexdens* (Table 2). The concentrations of the oil and its major components required to kill 50% of the *A. balzani* and *A. sexdens* workers 48 h after exposure by fumigation ranged from 2.18 to 5.72 µL·L^−1^ and 3.37 to 6.73 µL·L^−1^, respectively.

The monoterpenes linalool and sulcatyl acetate, when applied individually to *A. balzani*, were 1.6 and 1.7 times more toxic than the essential oil of *A. trilobata.* By contrast, *p*-cymene and limonene were less toxic to *A. balzani* than the essential oil. For *A. sexdens*, the limonene, linalool and sulcatyl acetate components showed toxicity similar to that of the essential oil of *A. trilobata*, and only the monoterpene *p*-cymene was 1.6 times more toxic than the essential oil (Table 2).

The tolerance of the ant species to the studied components varied. Comparing the two species, *A. sexdens* workers were more tolerant to the essential oil of *A. trilobata* and the components linalool and sulcatyl acetate, whereas *A. balzani* was more resistant to the monoterpene *p*-cymene. Limonene caused similar toxicity in both ant species (Table 2). In general, the greater susceptibility of *A. balzani* (9.26 mg body weight) compared with *A. sexdens* (15.09 mg body weight) may be related to its lower body volume and, consequently, higher specific area, which increases its exposure to the components [25,26,27]. Volatiles are known to penetrate through the spiracles; therefore, the chemical composition of the cuticle within such structures can also contribute to a greater or lesser penetration of the components present in the essential oil of *A. trilobata*, leading to variations in species tolerance [23].

Alternatively, the greater tolerance of *A. sexdens* compared with *A. balzani* may be associated with target site insensitivity or more efficient metabolism by detoxifying enzymes [23,25]. Considering that many mono- and sesquiterpenes present in the essential oil of *A. trilobata* act in insects by inhibiting acetylcholinesterase [28], changes in this enzyme and/or the speed with which it catalyzes the hydrolysis of the neurotransmitter acetylcholine may also be responsible for the higher tolerance of ants of the genus *Atta* compared with those of the genus *Acromyrmex*. Likewise, higher rates of metabolism for *A. trilobata* terpenes promoted by enzymes such as cytochrome P450-dependent monooxygenase, glutathione S-transferases and esterases may be responsible for the higher tolerance of ants of the genus *Atta* [23,25].

**Figure 3:**
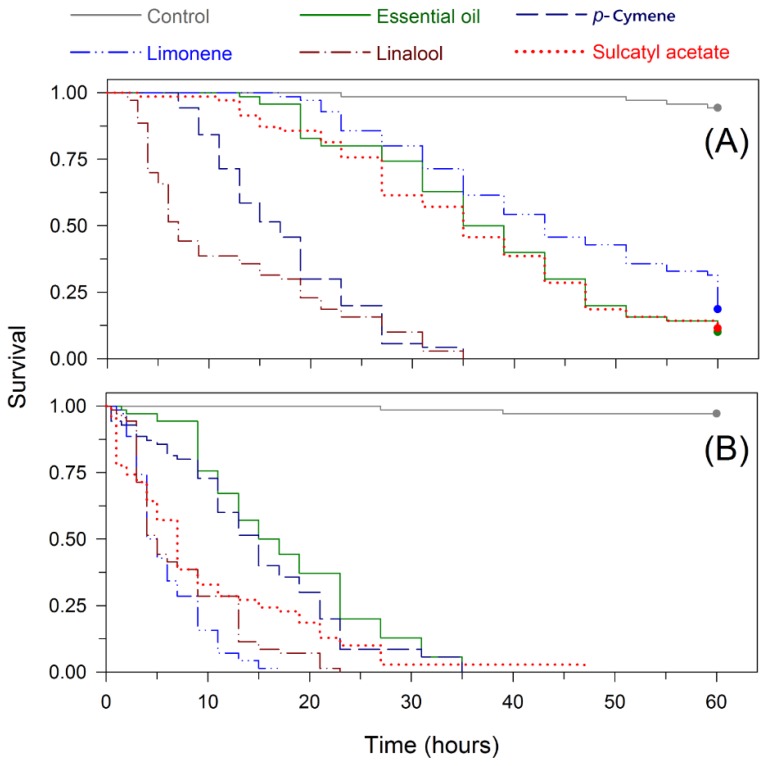
Survival curves of *Acromyrmex balzani* (**A**) and *Atta sexdens* (**B**) workers exposed to the LC_90_ of *Aristolochia trilobata* essential oil and its major components by fumigation. The LC_90_ was obtained in the toxicity bioassays.

The survival of *A. balzani* and *A. sexdens* workers exposed to the 90% lethal concentration (LC_90_) of the essential oil of *A. trilobata* and its individual major components was significantly reduced over time (by the log-rank test, χ^2^ = 463.2, df = 5 and *p* < 0.001 for *A. balzani*; χ^2^ = 338.0, df = 5 and *p* < 0.001 for *A. sexdens*) (Figure 3). The rapid insecticidal action of *A. trilobata* essential oil indicates that its toxicity is mainly mediated by the interaction of its constituents with target sites in the insect nervous system [29].

The effectiveness of insect fumigation with essential oils has been attributed to the uptake of oil through the spiracles [30]. This occurs because the spiracles are not an effective barrier against gas penetration. Thus, fumigation is the fastest and most efficient method for mono- and sesquiterpenes to reach the insect nervous system, which is the likely target site of these substances [31,32]. Certain essential oils have shown neurotoxic effects, causing hyperactivity, tremors and insect paralysis after exposure [29]. In fact, in the present study, it was observed that the essential oil of *A. trilobata* and its major components caused typical symptoms in ants of neurotoxic poisoning, such as the loss of motor coordination and the disruption of normal activities [33].

Previous studies have attributed the toxicity of the mono- and sesquiterpenes present in plant essential oils to several mechanisms, such as inhibition of the enzyme acetylcholinesterase; interference with the activity of octopamine, a neuromodulator found only in invertebrates [28]; interference with calcium channels modulated by γ-aminobutyric acid (GABA) [34]; and effects at vulnerable sites, such as cytochrome P450 [23]. However, it was not possible to determine with certainty which of these mechanisms operated in the insects evaluated in the present study; therefore, further studies on the mechanisms underlying the biochemical and pharmacological effects of the essential oil of *A. trilobata* and its major components are needed.

**Figure 4:**
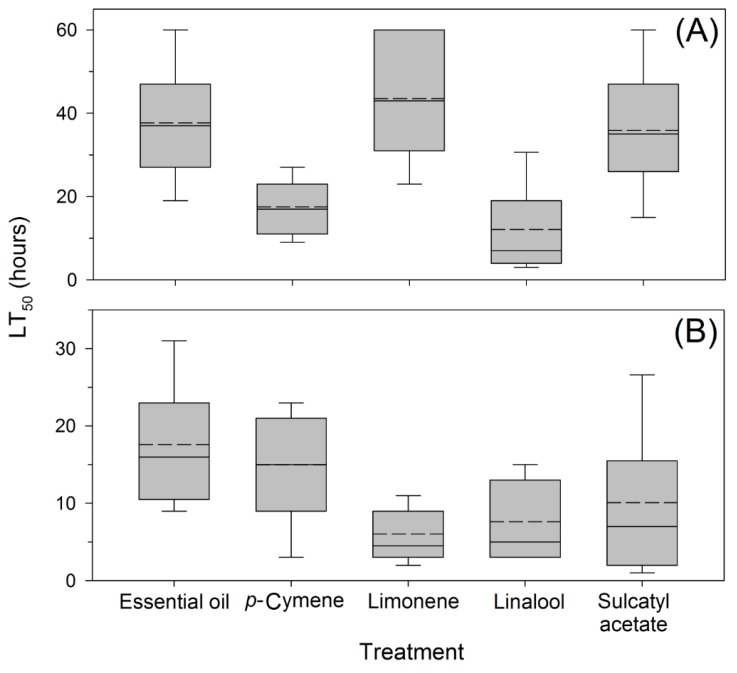
Mean LT for *Acromyrmex balzani* (**A**) and *Atta sexdens* (**B**) workers exposed by fumigation to the LC_90_ of the essential oil of *Aristolochia trilobata* and its major components. The boxes indicate the median and the dispersion (upper and lower quartiles). The dashed line represents the average of the LT_50_ values. The LC_90_ was obtained in the toxicity bioassays.

The survival of *A. balzani* and *A. sexdens* after 60 h of exposure exceeded 90% in the control group. In general, the essential oil of *A. trilobata* and its major components acted faster on *A. sexdens* than on *A. balzani* (Figure 3). The comparatively long time required by the oil and its components to kill *A. balzani* workers indicates a possible future use of these products in granulated baits for this species because there would be time for specimens to carry the toxic components into the nest [10,35]. By contrast, the rapid mortality of *A. sexdens* suggests a possible direct use in nests via a dry power or by thermal fogging. However, the practical use of these products requires the development of formulations suitable for each method of application [36]. The essential oil components *p*-cymene and linalool caused 100% mortality of both ant species in less than 35 h of exposure, whereas *A. trilobata* essential oil and limonene caused 100% mortality only in *A. sexdens*. Sulcatyl acetate also caused 100% mortality in *A. sexdens*; however, this effect required 47 h of exposure (Figure 3). These differences were reflected in the average time of survival (LT_50_) observed for each component (Figure 4). The average time of survival was not estimated for the insects in the control group because of the low mortality rate (<10%).

The essential oil of *A. trilobata* and its major components acted very quickly, killing half of the population of *A. sexdens* in less than 20 h. However, the essential oil of *A. trilobata*, limonene and sulcatyl acetate required 37.7, 43.5 and 35.8 h, respectively, to kill 50% of *A. balzani* (Figure 4).

### 2.4. Toxicity of Binary Mixtures

Bioassays using binary mixtures of the major components of the *A. trilobata* essential oil revealed that these components acted synergistically to cause toxicity in *A. balzani*. By contrast, the component combinations had an additive effect in *A. sexdens* (Table 3). The synergistic effect observed in *A. balzani* for the major components of the essential oil of *A. trilobata* can help explain the greater toxicity of the essential oil (i.e., a mixture of all components) to *A. balzani* than to *A. sexdens*.

The synergism that occurs between components of plant essential oils may result in a higher bioactivity than that observed when the components are used individually. Such effects are common for terpenes, which are hydrophobic compounds that exhibit synergistic effects with other components, solubilizing them and facilitating their dispersion through membranes [28]. Thus, this type of action increases the efficacy of these products, allowing the use of smaller amounts in a mixture to achieve satisfactory efficiency levels [37]. 

In addition to the interactions between the major components, there is still the possibility of other combinations showing synergistic effects. Minor components can act as synergists, increasing the efficacy of major components [10] and playing important roles in various oil properties, such as hypo- or hydrophilic attraction, fixation [8] and cell penetration [38].

By contrast, the binary mixtures had an additive effect in *A. sexdens*, with no potentiated effects when the essential oil components were combined. Studies have shown that complex interactions between the components of essential oils may vary with the circumstances [27,39]. Therefore, the additive effect of the essential oil components observed for *A. sexdens* may be explained by the different metabolic and physiological capabilities of the ant species when challenged with component mixtures.

### 2.5. Behavioral Bioassays

**Figure 5:**
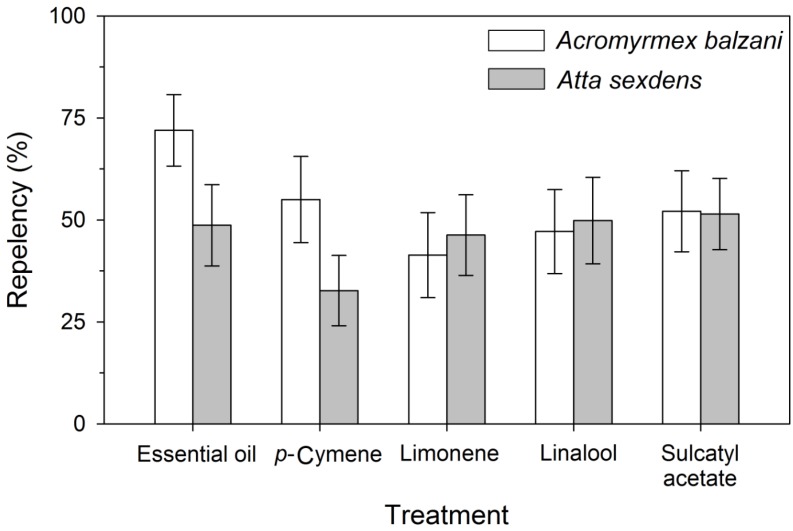
Repellency of the essential oil of *Aristolochia trilobata* and its major components to *Acromyrmex balzani* and *Atta sexdens* workers after a 15 min exposure to treated filter paper in glass dishes (9 cm diameter).

**Figure 6:**
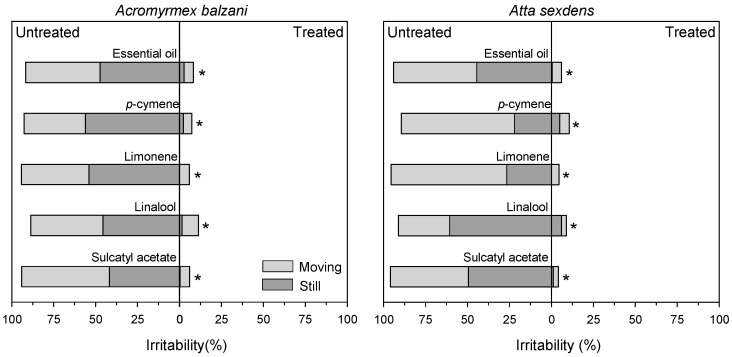
Irritability to the essential oil of *Aristolochia trilobata* and its major components of *Acromyrmex balzani* and *Atta sexdens* workers after a 15 min exposure in filter paper dishes (6 cm diameter). * Indicates a significant difference between the treated and untreated areas of the dish (Kruskal-Wallis test, *p* < 0.05).

The behavior of avoiding surfaces treated with *A. trilobata* essential oil and its major components was evaluated in two assays: a repellency (avoidance without previous contact) assay and an irritability (avoidance after contact) assay (Figure 5 and Figure 6).

The essential oil of *A. trilobata* and its major components were repellent to *A. balzani* and *A. sexdens*. On average, 53% and 46% of the experimental time elapsed before *A. balzani* and *A. sexdens* individuals first contacted the treated surface, respectively (Figure 5). There were no significant differences in the observed repellency among the treatments for *A. balzani* (Kruskal-Wallis: χ^2^ = 6.61; df = 4; *p* = 0.158) and *A. sexdens* (Kruskal-Wallis: χ^2^ = 2.6; df = 4; *p* = 0.167). The behavior of avoiding the components without contact confirms that the ants detect the essential oil and its constituents through chemical cues, avoiding the components when given the choice [40]. The repellency of plant secondary metabolites has been demonstrated for a variety of insect orders [10].

All the tested substances caused great irritability in *A. balzani* and *A. sexdens*. There were no significant differences in the amount of time the insects remained on the untreated side between *A. balzani* (Kruskal-Wallis: χ^2^ = 2.93; df = 4; *p* = 0.569) and *A. sexdens* (Kruskal-Wallis: χ^2^ = 6.81; df = 4; *p* = 0.146). In general, the insects spent more than 92% of the total time (immobile and moving) on the untreated side after contact (Figure 6). The irritability behavior confirms that the ants, besides detecting the essential oil and its constituents by chemical communication, also have single-pore sensilla responsible for detecting the oil components upon contact [40].

Understanding the behavior of ants upon their exposure to bioactive products is essential because leaf-cutting ants have resources to reduce the effect of chemical components harmful to them or their fungus. Chemical communication, olfactory sensitivity, learning capacity, selectivity and the production of antibiotic substances are the main behavioral factors that may hinder control [41]. Repellency and/or irritability effects may help prevent these behaviors of leaf-cutting ants in control areas.

## 3. Materials and Methods

### 3.1. Sampling Site and Plant Material

The plant material was collected in a mangrove area in the municipality of Pirambu, State of Sergipe (SE), Brazil (10°40′42″ S, 36°52′25″ W). The annual average temperature and rainfall in the region is 26 °C and 1650 mm, respectively, with the rainy season from March to August [42]. Stems (1 cm diameter) of the species *Aristolochia trilobata* were collected from plants in the vegetative growth stage between 9:00 a.m. and 12:00 p.m. from February to September 2014. The plant material was dried at 60 ± 1 °C for four days in a drying oven (Marconi MA 037) [43]. A voucher specimen was deposited in the herbarium of the Federal University of Sergipe (Universidade Federal de Sergipe—UFS), registration no. ASE 35.723. The herbarium is located in the Department of Biology (Departamento de Biologia), Biological Sciences and Health Center (Centro de Ciências Biológicas e da Saúde—CCBS), municipality of São Cristóvão, SE, Brazil, zip code 49100-000.

### 3.2. Essential oil Extraction and Analysis

The essential oil was obtained by hydrodistillation in a Clevenger-type apparatus [44]. Subsequently, the essential oil was separated from the aqueous phase and stored in an amber bottle in a freezer at −4 °C until used.

The analysis of the essential oil components was performed by a GC/MS/FID (GCMSQP2010 Ultra, Shimadzu Corporation, Kyoto, Japan) equipped with an AOC-20i autoinjector (Shimadzu). The separations were performed on 30 m, Rtx^®^-5MS Restek fused-silica capillary column (5% diphenyl–95% dimethylpolysiloxane) with a 0.25 mm internal diameter and 0.25 mm film thickness. Helium 5.0 was used as the carrier gas at a flow rate of 1.0 mL·min^−1^.

The injection temperature was 280 °C, and 1.0 μL (10 mg·mL^−1^) of sample was injected at a split ratio of 1:30. The oven temperature was programmed isothermally at 50 °C for 1.5 min, followed by an increase of 4 °C·min^−1^ until reaching 200 °C and then an increase of 10 °C·min^−1^ up to 300 °C, which was maintained for 5 min. For the GC/MS, the ionic capture detector impact energy was 70 eV. The fragments were analyzed by a quadrupolar system programmed to filter fragments/ions with *m*/*z* from 40 to 500 Da and detected by an electron multiplier. The data were processed with the aid of GCMS Post-run Analysis software (Labsolutions, Shimadzu).

The ionization process for GC/FID was conducted using the flame from hydrogen gas 5.0 (30 mL·min^−1^) and synthetic air (300 mL·min^−1^). The formed ions and the generated electric current were amplified and processed, and the data were analyzed with GCMS Post-run Analysis software (Labsolutions, Shimadzu).

The essential oil components were identified by a comparison of their retention times with those available in the literature [45]. The retention index was determined using the Van den Dool and Kratz (1963) equation [18], for a homologous series of *n*-alkanes (*n*C9-*n*C18). The essential oil components were also identified by comparing their mass spectra with the spectra available in the WILEY8, NIST107 and NIST21 equipment databases, which allow the comparison of mass spectral data sets and the use of a minimum similarity index of 80%.

### 3.3. Extraction of Major Components

**Scheme 1:**
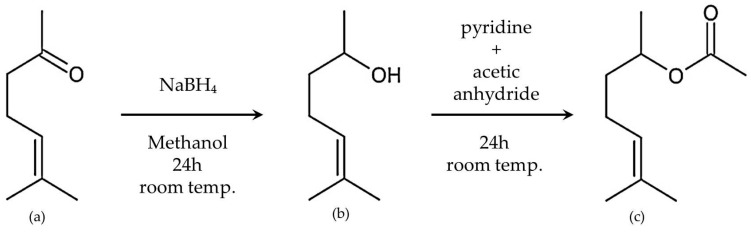
Scheme of sulcatyl acetate synthesis showing (**a**) sulcatone; (**b**) sulcatol; and (**c**) sulcatyl acetate.

The compounds individually composing at least 6% of *A. trilobata* essential oil were considered major components and included the following compounds: *p*-cymene, limonene, linalool and sulcatyl acetate. Chemical standards were purchased from Sigma-Aldrich^®^ (Steinheim, Germany), with the exception of (±)-sulcatyl acetate, which was synthesized by chemical reduction of 6-methyl-5-hepten-2-one (sulcatone, Scheme 1).

Briefly, sulcatone (500 mg, 3.97 mmol, Sigma-Aldrich^®^) was added into a 50 mL round-bottom flask and dissolved in methanol (10 mL) before NaBH_4_ (160 mg, 3.97 mmol) was added. The mixture was stirred magnetically at room temperature for 1 h. The reaction was then stopped by adding 1 mL of distilled water. The extraction of the product [(±)-6-methyl-5-hepten-2-ol (sulcatol)] was performed with ethyl acetate (20 mL), which was then removed by rotary evaporation. Sulcatol was completely dried with N_2,_ providing a 98% yield (500 mg, 3.91 mmol), and then treated pyridine (1 mL) and acetic anhydride (1 mL) for 24 h at room temperature. Subsequently, 10% HCl (4 mL) was added to the reaction mixture, and 2-acetyl-6-methyl-5-heptene (sulcatyl acetate) was extracted from the reaction mixture with ethyl acetate (20 mL). The solvent was removed by rotary evaporation. The sulcatyl acetate was completely dried with N_2_, and an 83% yield (550 mg, 3.24 mmol) was obtained.

The sulcatyl acetate was purified on freshly prepared TLC (thin layer chromatography) plates. Silica gel plates (1.0 mm thick) were prepared by evenly spreading ~30 g of Macherey-Nagel silica gel 60 in 80 mL of distilled water on glass sheets (20 cm × 20 cm). After evaporation of the water at room temperature, the TLC plates were activated in an oven at 110 °C for 30 min. For visualization, a solution of anisaldehyde in acid and ethanol (ethyl alcohol (90 mL) + sulfuric acid (5 mL) + anisaldehyde (5 mL) + acetic acid (1 mL) was used, followed by heating at 110 °C. For each preparative TLC plate, 300 mg of sulcatyl acetate was applied and eluted twice using as solvent hexane/ethyl acetate in proportion 9.5:0.5 (*v*/*v*). The analyses by nuclear magnetic resonance spectroscopy (^1^H- and ^13^C-NMR) (AVANCE III 400 NMR spectrometer, Bruker, Billerica, MA, USA) and infrared spectroscopy (IR, Spectrum BX Fourier Transform spectrometer, Perkin-Elmer, Waltham, MA, USA) were performed to confirm the structure of the major constituent, sulcatyl acetate (6-methyl-5-hepten-2-yl acetate) as described in [19].

### 3.4. Insects

*Acromyrmex balzani* and *Atta sexdens* workers were obtained directly from nests located at the UFS campus, municipality of São Cristóvão, SE, Brazil (10°54′ S, 37°04′ W). The ants were kept in nest fragments in round plastic containers (50 cm × 20 cm) at ambient conditions (25–27 °C and 60% ± 5% relative humidity) for 24 h prior to testing, and only distilled water was provided during this period.

### 3.5. Bioassays

The bioassays were conducted in a laboratory of the UFS in the municipality of São Cristóvão, SE, Brazil. All the treatment compounds were diluted in acetone solvent (Panreac, UV-IR-HPLC-GPC PAI-ACS, 99.9% purity). Preliminary tests showed that this use of acetone did not affect ant survival. The treatments consisted of *A. trilobata* essential oil and its following major components: *p*-cymene, limonene, linalool and sulcatyl acetate. Only acetone was used in the control groups.

#### 3.5.1. Routes of Exposure

The toxicity of *A. trilobata* essential oil and its major components was evaluated by two routes of exposure: contact by topical application and fumigation. To initially determine the treatment efficacy by these two routes of exposure, a dose of 10 µg·mg^−1^ was used in the contact bioassay, and a concentration of 10 μL·L^−1^ was used in the fumigation bioassay. Preliminary tests with other ant species indicated that this dose and this concentration are good indicators of efficiency. Further tests were conducted with the more efficient exposure route.

In both bioassays (contact and fumigation), the experimental design was completely randomized, with four replications. The experimental units were placed in a biochemical oxygen demand (BOD) incubator with a 25 ± 1 °C temperature, >70% relative humidity and a 12 h photoperiod. The specimens used were standardized by size for each ant species. For dose calculations, the mean body weight of *A. balzani* and *A. sexdens* was obtained by measuring the weight of 30 specimens with an analytical balance (AUW220D, Shimadzu, Kyoto, Japan) with a readability of 0.01 mg. The mortality was assessed 48 h after the bioassays were assembled. Any specimens that did not move or respond to stimulation were considered dead.

In the contact bioassays, each experimental unit consisted of a glass Petri dish (9 cm × 2 cm) lined by filter paper moistened with 0.5 mL of distilled water and containing seven workers. Preliminary tests indicated that these moisture conditions and seven ants per Petri dish favor long ant survival. The dishes with the ants were kept in a freezer at −4 °C for 1 min to reduce ant activity and allow topical application of the treatments. Preliminary tests indicated that this brief chill does not affect ant survival. Using a 10 μL microsyringe (Hamilton^®^, Renon, NV, USA) each individual was treated on the prothorax with 1 μL of acetone containing the essential oil of *A. trilobata*, one or two major oil components or no additional chemicals (control group). The dishes containing the treated insects were sealed with PVC film and placed in the BOD incubator.

In the fumigation bioassay, each replicate consisted of a glass container (250 mL) lined with filter paper moistened with 0.5 mL of distilled water and containing seven workers. Preliminary tests indicated that these moisture conditions and seven ants per container favor long ant survival. The treatments (essential oil of *A. trilobata* and its major components) were applied with a 10 μL Hamilton^®^ microsyringe to 1 cm^2^ pieces of filter paper (501.009, Unifil, Carvalhaes LTDA, Campo Limpo Paulista, São Paulo, Brazil). Each treated filter paper (1 cm^2^), the dispersion substrate for the volatiles, was secured to the bottom of a container lid using a thread. The thread kept the filter paper at the center of the glass container out of the reach of the ants, thus avoiding contact of the ants with the filter paper. A 10 μL aliquot of the solution of *A. trilobata* essential oil and of each major oil compound was used. The containers were sealed with a plastic lid and PVC film and then placed in the BOD incubator.

#### 3.5.2. Acute Toxicity Caused by Fumigation

Acute fumigation toxicity was analyzed using the LCs and LTs of the *A. trilobata* essential oil and its major components determined for the two ant species. The procedures used to determine the LC_50_ and LC_90_ were similar to those used in the fumigation bioassays (see Section 3.5.1). However, 14 and 10 concentrations of the *A. trilobata* essential oil and each major component, respectively, were used to determine the *A. balzani* and *A. sexdens* concentration-mortality curves.

The survival curves and LT_50_ were determined following the procedures used in the fumigation bioassay (see Section 3.5.1). However, only one concentration was used (the LC_90_ determined by the bioassay explained above for each treatment), with 10 repetitions. The mortality was assessed every 30 min during the first 2 h of the experiment, every 60 min up to 7 h, every 120 min up to 23 h and, subsequently, every 240 min up to 60 h.

#### 3.5.3. Toxicity of the Binary Mixtures

The acute fumigation toxicity of binary mixtures containing the major components of *A. trilobata* oil (*p*-cymene, limonene, linalool and sulcatyl acetate) were determined by procedures similar to those used in the fumigation bioassay (see Section 3.5.1).

Initially, the LC_50_ values of the most effective components determined in the fumigation bioassay (see Table 2) were used to establish the concentrations appropriate to bioassay the binary mixtures. The pairs were formed using an efficiency rank based on the lower and upper concentration limits of the LC_50_ confidence intervals of the most effective components in 1:1 mixtures.

The major components were applied individually and in combination at those concentrations on each ant species for comparison. The observed and expected mortalities were compared, and the effects of the binary mixtures were classified as additive, synergistic or antagonistic.

#### 3.5.4. Behavioral Bioassays

The behavioral effects of the *A. trilobata* essential oil and its major components on *A. balzani* and *A. sexdens* workers were separated into repellency (avoidance without previous contact) and irritability (avoidance after contact) [46].

The bioassays were performed in glass Petri dishes (9 cm× 2 cm) lined with filter paper, which was divided into treated and untreated (acetone only) halves. The treated halves of the papers received 0.4 mL of the treatment solutions at a concentration of 1%, which was insufficient to kill the specimens during the study period. The treated and untreated paper halves were placed in an exhaust hood for 5 min for solvent evaporation after affixing them to the bottom of the dishes with double-sided tape.

A single adult *A. balzani* or *A. sexdens* worker was placed in the center of the Petri dish. For each combination of species × treatment, 20 replicates were used, totaling 200 Petri dishes. The observations consisted of the continuous recording for 15 min of the amount of time that the ant spent both stationary and mobile on each side of the dish and that elapsed before the ant first contacted the treated area. The ant was considered to have made contact when it remained longer than 1 s in the treated area.

The strength of repellency was determined by measuring the time required for the ant to contact the treated half of the filter paper, with longer time indicating greater treatment repellency. By contrast, irritability was determining by measuring the amount of time the ant remained (still and/or moving) on each side of the plate after the first contact, with longer time spent on the untreated half of the filter paper indicating greater irritability.

### 3.6. Statistical Analysis

The mortality results for the bioassays concerning routes of exposure, fumigation acute toxicities for the determination of LCs and binary mixture toxicities were corrected for the mortality rate in the control group using Abbott’s formula [47].

To determine efficiencies for the routes of exposure, the present study used an insecticide registration criterion of the Ministry of Agriculture, Livestock and Food Supply of Brazil (Ministério da Agricultura e Pecuária do Brasil—MAPA). The MAPA regulation states that an insecticide must result in a minimum mortality of 80% [24].

Probit analyses were performed to determine the concentration-mortality curves for the *A. trilobata* essential oil and its major components in each ant species. The curves with a probability greater than 0.05 for the acceptance of the null hypothesis by a χ^2^ test were accepted. The LC_50_ and LC_90_ and their respective 95% confidence intervals were obtained using the accepted curves and Statistical Analysis System (SAS) software [48]. The LCs were compared and considered to differ significantly from one another when their 95% confidence intervals (CIs95) did not overlap.

The results of the LT bioassays were subjected to survival analysis using SAS software [48]. This nonparametric analysis allows the estimation of survival curves obtained by the Kaplan-Meier estimator using the insect survival rate from the beginning to the end of the experiment. The times required for each of the treatments to kill 50% of each ant species were determined.

In the binary mixture bioassays, the expected mortalities were calculated according to the following formula described by [9]. E=Oa+Ob (1−Oa), where *E* is the expected mortality, and *O*a and *O*b are the observed mortality caused by the pure components.

The effects of the binary mixtures were classified by comparisons between the calculated χ^2^ and the tabulated χ^2^ (χ^2^_tab_ = 3.84; df = 1; α = 0.05). χ^2^ was calculated using the following formula:

$$\chi^{2}=\frac{{\left(Om-E\right)}^{2}}{E},$$

where *O*m is the observed mortality of the binary mixture. If χ^2^_cal_ < 3.84 for the pair analyzed, there is an additive affect (also called a noneffect). If χ^2^_cal_ > 3.84 for the pair analyzed, there is a synergistic or antagonistic effect. In the latter case, the expected and observed mortalities of the binary mixtures should be noted.

The behavioral bioassay data were analyzed for conformity with the assumptions of normality and homogeneity of variance using SAS software [48]. Subsequently, the repellency and irritability data were subjected to the nonparametric Kruskal-Wallis test, followed by Wilcoxon’s test at *p* < 0.05 using SAS software [48].

## 4. Conclusions

*A. trilobata* essential oil and its major components, *p*-cymene, limonene, linalool and sulcatyl acetate, show rapid and high fumigation toxicity to the leaf-cutting ant species *A. balzani* and *A. sexdens*. All the treatments caused not only significant reductions in the survival of both ant species but also strong behavioral effects of repellency and irritability. For *A. balzani*, the major components interacted synergistically, enabling the use of lower doses to control this species. Further studies are needed to elucidate the effects of these components in natural environments. The present study demonstrates the potential of the essential oil of *A. trilobata* and its major components as alternatives to the use of conventional insecticides and as promising sources of new molecules with insecticidal activity.

## Figures and Tables

**Table 1:** Composition of the essential oil of *Aristolochia trilobata* characterized by GC/MS/FID.

Component	Retention Time (min) ^1^	Retention Index ^1^	Peak Area (%) ^2^
Tricyclene	8.985	929	1.88 ± 0.02
Camphene	9.469	944	3.24 ± 0.012
β-Pinene	10.353	973	1.05 ± 0.02
Myrcene	10.681	984	0.68 ± 0.03
6-Methyl-5-hepten-2-ol	10.728	985	0.64 ± 0.08
*p*-Cymene	11.859	1020	10.41 ± 0.03
Limonene	12.021	1025	24.80 ± 0.26
(*Z*)-β-Ocimene	12.546	1041	5.27 ± 0.42
Linalool	14.257	1092	9.51 ± 0.05
Sulcatyl acetate	15.220	1122	25.64 ± 0.45
Borneol	16.568	1165	0.64 ± 0.05
α-Terpineol	17.328	1189	0.28 ± 0.02
Bornyl acetate	20.222	1283	0.79 ± 0.007
β-Elemene	23.345	1391	0.30 ± 0.012
(*E*)-Caryophyllene	24.260	1424	1.15 ± 0.03
Aromadendrene	24.633	1438	0.27 ± 0.01
Allo-Aromadendrene	25.415	1467	0.27 ± 0.0
Germacrene D	25.922	1485	0.70 ± 0.007
Bicyclogermacrene	26.343	1501	1.52 ± 0.02
δ-Cadinene	26.933	1524	0.40 ± 0.06
Spathulenol	28.508	1586	3.04 ± 0.10
Globulol	28.695	1594	4.29 ± 0.13
Viridiflorol	28.903	1602	0.49 ± 0.03
Isospathulenol	29.971	1646	0.94 ± 0.05
α-Cadinol	30.348	1662	0.55 ± 0.05
Monoterpenes (%)			84.83
Sesquiterpenes (%)			13.89
Total (%)			98.72

^1^ Retention index calculated using the Van den Dool and Kratz (1963) equation [18] relative to a homologous series of *n*-alkanes (*n*C9-*n*C18). ^2^ Values (±SEM) for the content of components obtained by averaging three different determinations obtained by GC/MS/FID.

**Table 2:** Toxicity of *Aristolochia trilobata* essential oil and its major components to *Acromyrmex balzani* and *Atta sexdens* workers measured 48 h after exposure by fumigation.

Treatment	N° of Insects	LC_50_ (95% CI) (µL·L^−1^)	LC_90_ (95% CI) (µL·L^−1^)	Slope	χ^2^	*p-*Value
*Acromyrmex balzani*						
Essential oil	239	3.76 (3.46–4.13)	9.33 (7.65–12.68)	3.24	0.15	0.98
*p*-Cymene	490	4.96 (4.41–5.56)	16.53 (13.08–22.83)	2.44	13.73	0.05
Limonene	546	5.72 (5.32– 6.14)	10.34 (9.39–11.68)	4.98	0.61	0.74
Linalool	238	2.40 (1.92–3.21)	17.51 (10.35–40.99)	1.48	3.55	0.81
Sulcatyl acetate	266	2.18 (2.06–2.30)	3.38 (3.11–3.76)	6.75	1.67	0.56
*Atta sexdens*						
Essential oil	476	5.48 (4.66–6.34)	20.94 (16.78–28.33)	2.20	0.24	0.88
*p*-Cymene	420	3.37 (2.69–4.04)	17.71 (13.38–26.86)	1.77	0.36	0.83
Limonene	420	5.38 (4.56–6.28)	28.79 (21.33–44.74)	1.75	5.18	0.15
Linalool	308	5.34 (4.67–6.28)	19.88 (14.24–34.61)	2.24	1.72	0.57
Sulcatyl acetate	330	6.73 (5.41–10.40)	20.75 (12.56–60.72)	2.61	1.94	0.61

**Table 3:** Effect of binary mixtures of the major components composing *Aristolochia trilobata* essential oil on *Acromyrmex balzani* and *Atta sexdens* workers measured 48 h after exposure by fumigation.

Component A	Component B	Conc. ^1^ (µL·L^−1^)	Mortality (%)				χ^2^	Effect
			Pure Component		Binary Mixture			
			Observed A	Observed B	Expected	Observed		
*Acromyrmex balzani*								
Sulcatyl acetate	Linalool	2.30 + 2.30	21.43	19.64	36.86	67.86	26.06	Synergistic
Sulcatyl acetate	Linalool	2.06 + 2.06	7.14	10.71	17.09	39.29	28.82	Synergistic
Sulcatyl acetate	*p*-Cymene	2.30 + 2.30	21.43	25.00	41.07	62.50	11.18	Synergistic
Sulcatyl acetate	*p*-Cymene	2.06 + 2.06	7.14	5.36	12.12	23.21	10.16	Synergistic
Sulcatyl acetate	Limonene	2.30 + 2.30	21.43	3.57	24.23	46.43	20.32	Synergistic
Sulcatyl acetate	Limonene	2.06 + 2.06	7.14	3.57	10.46	32.14	44.95	Synergistic
Linalool	*p*-Cymene	3.21 + 3.21	16.07	23.21	35.55	67.86	29.35	Synergistic
Linalool	*p*-Cymene	1.92 + 1.92	10.71	3.57	13.90	30.36	19.47	Synergistic
Linalool	Limonene	3.21 + 3.21	16.07	5.36	20.57	66.07	100.67	Synergistic
Linalool	Limonene	1.92 + 1.92	10.71	5.36	15.50	26.79	8.22	Synergistic
*p*-Cymene	Limonene	5.56 + 5.56	51.79	14.29	58.67	87.50	14.16	Synergistic
*p*-Cymene	Limonene	4.41 + 4.41	44.64	17.86	54.53	82.14	13.99	Synergistic
*Atta sexdens*								
*p*-Cymene	Linalool	4.04 + 4.04	33.93	50.00	66.96	60.71	0.58	Additive
*p*-Cymene	Linalool	2.69 + 2.69	19.64	39.29	51.21	44.64	0.84	Additive
*p*-Cymene	Limonene	4.04 + 4.04	33.93	16.07	44.55	33.93	2.53	Additive
*p*-Cymene	Limonene	2.69 + 2.69	19.64	8.93	26.82	19.64	1.92	Additive
*p*-Cymene	Sulcatyl acetate	4.04 + 4.04	33.93	67.86	78.76	92.86	2.52	Additive
*p*-Cymene	Sulcatyl acetate	2.69 + 2.69	19.64	17.86	33.99	44.64	3.34	Additive
Linalool	Limonene	6.28 + 6.28	55.36	50.00	77.68	87.50	1.24	Additive
Linalool	Limonene	4.67 + 4.67	42.86	23.21	56.12	60.71	0.38	Additive
Linalool	Sulcatyl acetate	6.28 + 6.28	55.36	75.00	88.84	98.21	0.99	Additive
Linalool	Sulcatyl acetate	4.67 + 4.67	42.86	35.71	63.27	75.00	2.18	Additive
Limonene	Sulcatyl acetate	6.28 + 6.28	39.29	82.14	89.16	91.07	0.04	Additive
Limonene	Sulcatyl acetate	4.56 + 4.56	26.79	48.21	62.09	76.79	3.48	Additive

^1^ LC_50_ confidence intervals for the components used in the toxicity bioassays. The pairs were formed using an efficiency rank based on the lower and upper concentration limits of the LC_50_ confidence intervals of the most effective components in mixtures. For *Acromyrmex balzani*, sulcatyl acetate was the most toxic compound, followed in decreasing order of toxicity by linalool, *p*-cymene and limonene. For *Atta sexdens* the decreasing order of toxicity was: *p*-cymene, followed by linalool, limonene and sulcatyl acetate.
